# CRISPR/Cas9-Mediated Editing of *AGAMOUS-like* Genes Results in a Late-Bolting Phenotype in Chinese Cabbage (*Brassica rapa* ssp. *pekinensis*)

**DOI:** 10.3390/ijms232315009

**Published:** 2022-11-30

**Authors:** Yun-Hee Shin, Hyun-Min Lee, Young-Doo Park

**Affiliations:** Department of Horticultural Biotechnology, Kyung Hee University, 1732 Deogyoung-daero, Giheung-gu, Yongin-si 17104, Gyeonggi-do, Republic of Korea

**Keywords:** *AGL19* gene, *AGL24* gene, *Brassica rapa*, CRISPR/Cas9, late bolting

## Abstract

Due to the sudden change in temperature in spring, Chinese cabbage, a leafy vegetable cultivated for consumption, loses its commercial value due to the onset of bolting—the phenomenon of switching from vegetative to reproductive growth. In this study, we applied clustered regularly interspaced short palindromic repeats/(CRISPR)-associated system 9 (CRISPR/Cas9) technology to analyze *AGAMOUS-like* genes. We performed functional analysis of *AGL19* and *AGL24* genes related to bolting and flowering using CRISPR/Cas9-mediated Chinese cabbage transformation. Single-guide RNA (sgRNA) sequences were created with a low off-targeting probability to construct gene-editing vectors. *Agrobacterium*-mediated transformation was conducted, and tentative E_0_ *AGL*-edited lines were analyzed using molecular biotechnological methods. Two *AGL19*-edited lines with nucleotide sequence mutations in the target sequence of the *AGL19* genes and four *AGL24*-edited lines with nucleotide sequence mutations in the target sequence of the *AGL24* genes showed particularly late bolting compared to the inbred line ‘CT001.’ Generational progression using bud pollination obtained T-DNA-free E_1_ *AGL*-edited lines, which also showed late bolting. The loss of function of the AGL protein was caused by the occurrence of an indel mutation in the *AGL19* and *AGL24* genes, which results in an early stop codon. Furthermore, frameshift mutations led to structural changes and the introduction of an early stop codon in the AGL19 and AGL24 proteins. Our results indicate that CRISPR/Cas9-mediated editing of *AGAMOUS-like* genes results in a late-bolting phenotype and that CRISPR/Cas9 is a useful technology for analyzing gene function in Chinese cabbage (*Brassica rapa* ssp. *pekinensis*).

## 1. Introduction

Crops belonging to the genus *Brassica*, including rapeseed, cabbage, and Chinese cabbage, are cultivated on a large scale worldwide. Chinese cabbage is one of the three major vegetable crops cultivated in Korea. Chinese cabbage is an important crop that supplies vitamins, calcium, and minerals, and new varieties are currently being developed [1]. Chinese cabbage is actively cultivated and consumed in Korea and is known to grow well in cool temperatures. However, Chinese cabbage may bolt early due to rise in temperatures in early spring as a result of climate change in Korea [2]. The bolting phenomenon may occur in these plants in response to low temperatures. The commercial availability of Chinese cabbage, a vegetable cultivated for leaf consumption, is particularly adversely affected by the change from the vegetative to the reproductive growth stage [3,4,5].

The ABC model is a scientific model related to flower development comprising three classes. It induces sexual reproduction and floral organ emergence by regulating floral-related gene expression patterns. Recently, the ABC model has been expanded to the ABCDE model and studied for various crops. Class A is composed of floral meristem genes, and class B genes determine the construction of petals and stamens [6]. Although the expression of class B and C genes is reduced in *lfy-6*-null mutants, their expression is not abolished [7], indicating that other factors may also contribute to the activation of floral homeotic genes.

Class C includes *AG* genes. Previous studies using loss-of-function and gain-of-function analyses have revealed that *AGAMOUS-like 19* (*AGL19*) and *AGAMOUS-like 24* (*AGL24*) are flowering-related genes. Transgenic plants overexpressing the *AGL19* gene in the *FLC*-independent pathway appeared to flower earlier than the wild type [8]. The *agl19* mutants showed slightly late flowering compared to the wild type on short days. Likewise, the *agl24* mutants appeared to be late flowering, and transgenic plants overexpressing the *AGL24* gene showed early flowering [9]. Yeast two-hybrid assays revealed that *AGL19* not only interacts with *AGL24* but also indirectly interacts with *SUPPRESSOR OF OVEREXPRESSION OF CO 1* (*SOC1*) in *Brassica oleracea* [10]. Several key flowering-related genes, including *FLOWERING LOCUS C* (*FLC*), *VERNALIZATION 1* (*VRN1*), and *SOC1*, have been the focus of studies on bolting and flowering in Chinese cabbage [5,11,12]. Therefore, it is necessary to study *AGAMOUS-like* genes involved in bolting and flowering that act independently or are correlated with important regulatory genes.

Current research is focused on inculcating crops with useful characteristics to overcome the unfavorable external environment using various molecular biological technologies. For example, RNA interference (RNAi) has been used to develop insect-resistant transgenic plants. Transgenic tomato plants expressing artificial microRNA gained improved resistance to *Myzus persicae* on silencing the *acetylcholinesterase 1* (*Ace 1*) gene [13]. Transgenic Chinese cabbage producing siRNA gained enhanced resistance to *Tetranychus urticae* on silencing the *coatomer protein complex subunit 2* (*COPB2*) gene [14]. Insect-feeding assays conducted with transgenic plants showed resistance against pests and a high insect mortality rate. Transgenic plants capable of surviving abiotic stresses, such as salt, drought, and heat, have also been developed and analyzed. Transgenic tomato plants bearing *PpNAC56*, a transcription factor involved in numerous plant growth processes, are more resistant to heat stress than are the wild type [15]. Salt tolerance in transgenic Chinese white poplar has been improved by over-expression of the *mannitol-1-phosphate dehydrogenase* (*mtlD*) gene [16]. The only limitation to this technique is the insertion of T-DNA into the plant genome. However, advances in molecular biological technology, especially clustered regularly interspaced short palindromic repeats/(CRISPR)-associated system (CRISPR/Cas) technology, has allowed plant characteristics to be manipulated without having to maintain the T-DNA insertion [17].

Previous studies have shown that CRISPR/Cas technology allows T-DNA segregation via next-generation progression after target gene mutation, resulting in the desired traits being displayed in T-DNA-free plants. For example, salinity tolerance has been observed in *OsRR22*-edited T_1_ rice plants [18], and *CaERF28*-edited T_2_ chili plants showed improved anthracnose resistance [19].

CRISPR/Cas9 is an acquired immune system inherent in prokaryotes that recognizes and cleaves external viruses [20]. The operating mechanism of CRISPR/Cas9 is characterized as follows: crRNAs with complementary bases for target genes bind to tracrRNAs and combine with Cas9 proteins. This induces Cas9 to recognize and cut target genes [21]. In other words, using Cas9 and guide RNA, certain areas of DNA can be recognized and cut. The cut DNA is then recovered via various DNA repair pathways [22]. The DNA repair pathways can be divided into two categories. The first is homology-directed repair (HDR), in which DNA is accurately restored using homologous DNA manipulation as a template [23]. The second is non-homologous end joining (NHEJ), which induces insertion and deletion mutations. The addition or removal of several bases leads to a knock-out in which the codon composition of the gene is shifted and its function is disrupted [24]. A frameshift in the target gene inhibits the production of normal proteins by either creating a stop codon at an abnormal location of the mRNA or inhibiting the expression of proteins through mRNA [25].

In this study, using the CRISPR/Cas9 system, we confirmed that *AGAMOUS-like 19 (AGL19)* and *AGL24* genes [8,26] are related to bolting and flowering in Chinese cabbage. The CRISPR/Cas9 system targeted *AGL* genes, and late bolting was observed in the edited lines.

## 2. Results

### 2.1. Vector Construction and Agrobacterium-Mediated Transformation of Chinese Cabbage

**Figure 1:**
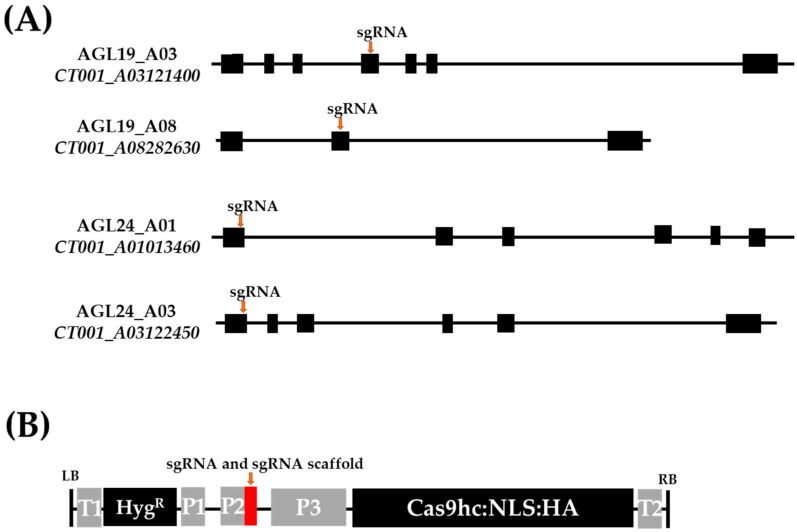
Gene structure analysis and construction of gene-editing vector. (**A**) Genomic structure of *AGL* and each homologous gene. Black box, exon regions; black line, intron regions; orange arrow, target site of single-guide RNAs (sgRNAs). (**B**) Schematic representation of the T-DNA in gene-editing vectors. LB, left border; T1, NOS terminator; Hyg^R^, hygromycin resistance gene; P1, NOS promoter; P2, Arabidopsis U6 promoter; P3, 35S promoter; Cas9hc:NLS:HA, human-codon-optimized Cas9 with the nuclear localization signal and an HA epitope; T2, 35S terminator; RB, right border. Red box, sgRNA and sgRNA scaffold.

Gene structure analysis was performed to design the single-guide RNA (sgRNA) sequence targeting the *AGL19* (*CT001_A03121400* and *CT001_A08282630*) and *AGL24* (*CT001_A03122450* and *CT001_A01013460*) genes of the inbred line ‘CT001.’ The *CT001_A03121400* gene, comprising seven exons, was positioned on the A03 chromosome, and the *CT001_A08282630* gene, comprising three exons, was identified on the A08 chromosome. *CT001_A03122450* was located on the A03 chromosome, and *CT001_A01013460* was located on the A01 chromosome. *CT001_A03122450* and *CT001_A01013460* contained six exons. sgRNAs were designed to target the exonic regions of each gene (Figure 1A). To avoid sequence editing other genes present in the genome of the inbred line ‘CT001,’ we generated sgRNA based on high specificity. By analyzing the sgRNA sequence showing the off-target effect, it was confirmed whether the region was a genetic or an intergenic one. In addition, the number of cases that can occur in the NGG nucleotide sequence (N can be A, T, G, or C) was confirmed. These sgRNAs were able to target specific regions without off-target effects. pAGL19 (A1) and pAGL24 (A2) editing vectors were constructed based on the pHAtC gene-editing vector and transformed with *Agrobacterium tumefaciens* LBA4404.

### 2.2. Selection and Bolting Time Analysis of E_0_ AGL-Edited Chinese Cabbage Lines

**Figure 2:**
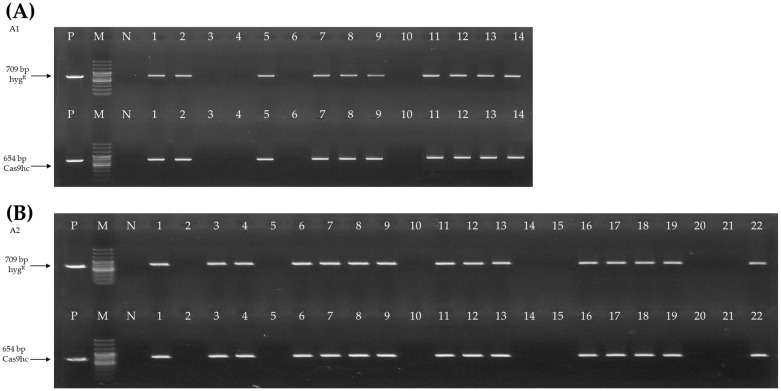
Selection of E_0_ *AGAMOUS-like (AGL)*-edited lines by polymerase chain reaction (PCR) analysis. (**A**) PCR analysis with hyg^R^ and Cas9hc primer sets of E_0_ *AGL19*-edited lines. (**B**) PCR analysis with hyg^R^ and Cas9hc primer sets of E_0_ *AGL24*-edited lines. The 709 bp and 654 bp PCR amplicons are indicated with an arrow. P, positive control; M, 100 bp DNA ladder; N, negative control; numbering lane, tentative gene-edited lines.

Tentative E_0_ *AGL*-edited lines were generated by transformation of Chinese cabbage. We obtained 14 and 22 tentative E_0_ lines derived from A1 and A2 editing vectors, respectively. To select the T-DNA-inserted line, two primer sets were designed using a partial sequence of hyg^R^ and Cas9hc:NLS:HA (Cas9hc) for PCR analysis (Table S1 in Supplementary Materials). Of the 14 A1 and 22 A2 E_0_ lines, 14 and 15 lines appeared as target amplicons, respectively (Figure 2).

**Figure 3:**
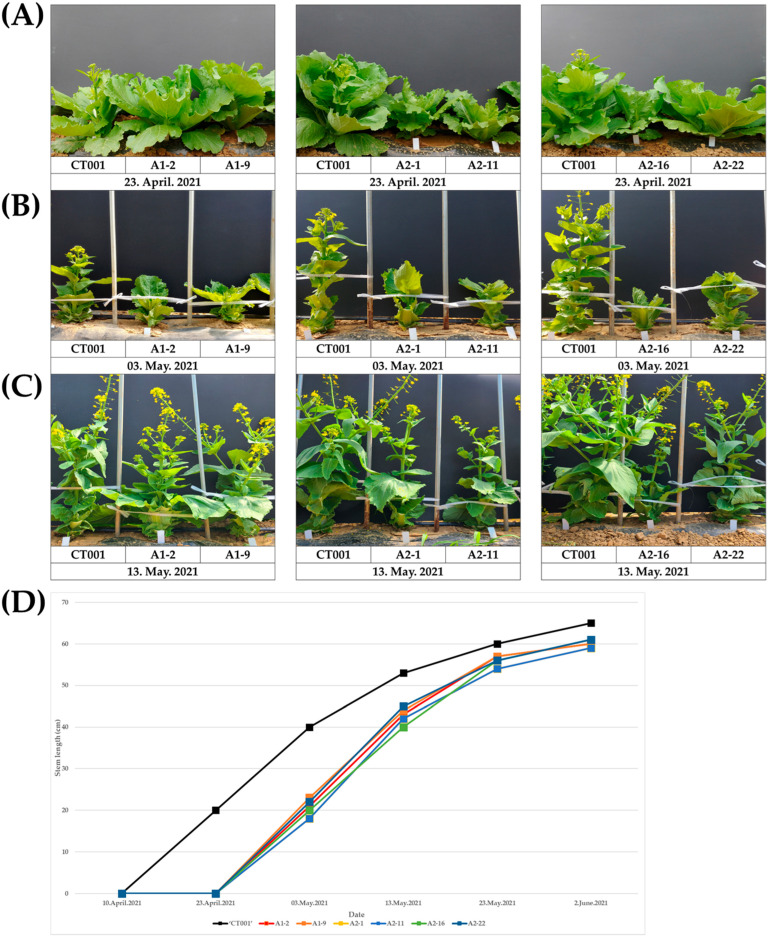
Observation of bolting time and stem length of inbred line ‘CT001′ and E_0_ *AGL*-edited lines. (**A**) Beginning stage of bolting. (**B**) Early stage of bolting. (**C**) Middle stage of bolting. Left, inbred line ‘CT001′; middle and right, E_0_ *AGAMOUS-like (AGL)*-edited lines. (**D**) Stem length of inbred line ‘CT001′ and E_0_ *AGL*-edited lines. Stem length gained from beginning to end of bolting in each line. The stem length was measured from the ground to the shoot apex.

To compare the bolting time of ‘CT001′ and the selected E_0_ *AGL*-edited lines, the date of the beginning of bolting was counted after vernalization. E_0_ lines usually showed late bolting; especially, A1-2, A1-9, A2-1, A2-11, A2-16, and A2-22 showed a significantly late-bolting phenotype (Figure 3A–C). When E_0_ *AGL*-edited lines appeared bolted, the stem length was 20.3 cm on average, and the stem length of ‘CT001′ showing faster bolting was 40 cm. On average, E_0_ *AGL*-edited lines bolted 10 days later than ‘CT001’.

**Figure 4:**
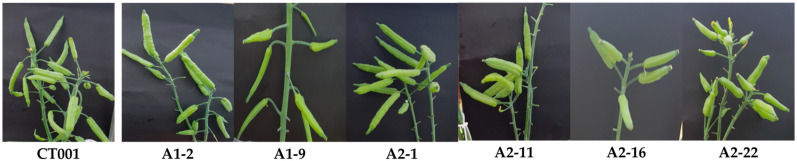
Pod formation following bud pollination in ‘CT001′ inbred line and E_0_ *AGAMOUS-like (AGL)*-edited lines. No difference in pod shape and seed formation was observed between inbred and E_0_ *AGL*-edited lines.

There was no difference in the growth process of the inbred ‘CT001′ and E_0_ *AGL*-edited lines, and when the bolting stage was completed, the stem length was restored to 60 cm on average, which was similar to the stem length of the ‘CT001′ line (Figure 3D). Based on the bolting time analysis, the selected E_0_ *AGL*-edited lines were self-pollinated and advanced to the E_1_ generation. The E_0_ *AGL*-edited lines formed normal pods and seeds (Figure 4), and no significant difference in the pod shape and germination rate of seeds between the ‘CT001′ and E_1_ *AGL*-edited lines was detected.

### 2.3. Mutagenesis of AGL Induced by CRISPR/Cas9 in E_0_ AGL-Edited Lines

**Figure 5:**
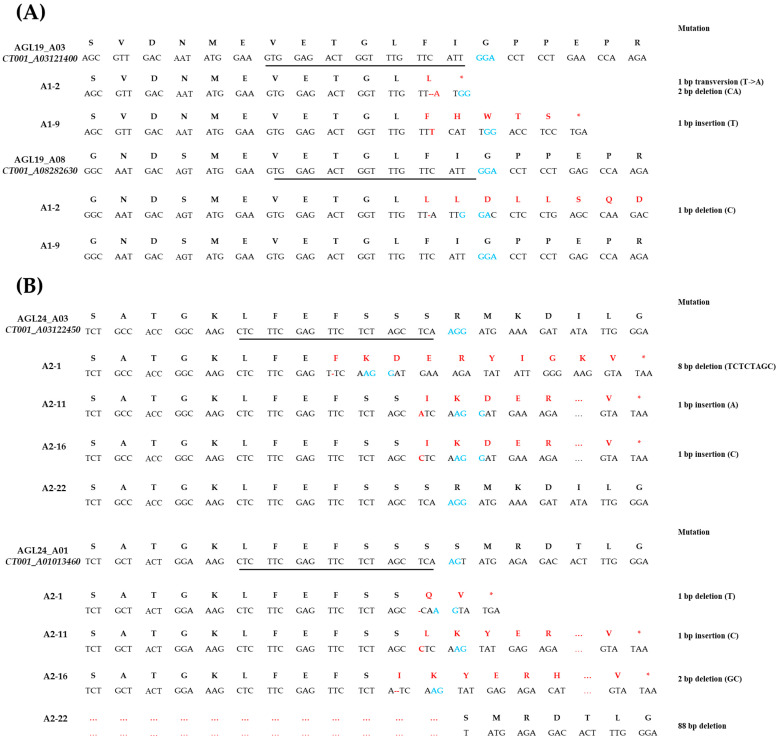
Mutagenesis patterns induced by clustered regularly interspaced short palindromic repeats (CRISPR)/Cas9 in E_0_ gene-edited lines. (**A**) Comparison of each nucleic and amino acid sequence of *AGL19* genes (*CT001_A03121400* and *CT001_A08282630*) in A1-2 and A1-9 lines. (**B**) Comparison of each nucleic and amino acid sequence of *AGL24* genes (*CT001_A03122450* and *CT001_A01013460*) in A2-1, A2-11, A2-16, and A2-22 lines. The underline indicates single-guide RNA (sgRNA), and the blue font indicates the protospacer adjacent motif (PAM) sequence; the red font represents the presence of sequence mutations and the resulting change in the amino acid sequence; red star, stop codon.

After bolting occurred, the E_0_ *AGL*-edited lines were analyzed for target gene mutation patterns. Some of the E_0_ *AGL*-edited lines showing late bolting were analyzed using PCR and RT-PCR analysis with gene-specific primers (Table S1 in Supplementary Materials). PCR and RT-PCR amplicons were analyzed using Sanger sequencing. As a result, nucleotide insertion and deletion were identified in E_0_ *AGL*-edited lines (Figure 5).

In the *CT001_A03121400* gene of the E_0_ A1-2 line, a single nucleotide that exhibited transversion was observed and two nucleotides were deleted. The *CT001_A08282630* gene was also targeted, and a sequence containing a single-nucleotide deletion was mutated and a frameshift occurred (Figure 5A). The E_0_ A1-9 line was only mutated in the *CT001_A03121400* gene, which included a thymine insertion 4 bp upstream of the protospacer adjacent motif (PAM) sequence and generation of a stop codon. Similarly, an 8 bp sequence was deleted upstream of the PAM sequence in the *CT001_A03122450* gene of the E_0_ A2-1 line. Five amino acid sequences were mutated, and the stop codon appeared early (Figure 5B). The thymine 3 bp upstream of the PAM sequence in the *CT001_A01013460* gene of the E_0_ A2-1 line was removed, and a stop codon emerged after the insertion of two amino acids (Figure 5B). In addition, a single nucleotide was inserted into the *CT001_A03122450* gene, which introduced an amino acid mutation that changed serine to isoleucine, resulting in a frameshift in the E_0_ A2-11 line. The formation of a stop codon was also observed (Figure 5B). A single nucleotide was inserted in the *CT001_A01013460* gene of the E_0_ A2-11 line, changing it from serine to leucine. Similarly, a stop codon was observed. Cytosine was inserted near the PAM sequence in the *CT001_A03122450* gene of the E_0_ A2-16 line, and a frameshift occurred (Figure 5B). Deletion of 2 bp in the *CT001_A01013460* gene of the E_0_ A2-16 line occurred, resulting in 9 amino acids produced by the frameshift, followed by a stop codon (Figure 5B). No mutation was detected in the *CT001_A03122450* gene of the E_0_ A2-22 line, but a large deletion was identified in the *CT001_A01013460* gene (Figure 5B). These mutations resulted in abnormal gene function and caused AGL-edited lines to flower later than the control line, ‘CT001’.

### 2.4. T-DNA Copy Number and Flanking Site Sequence Analysis of Late-Bolting E_0_ AGL-Edited Lines

**Figure 6:**
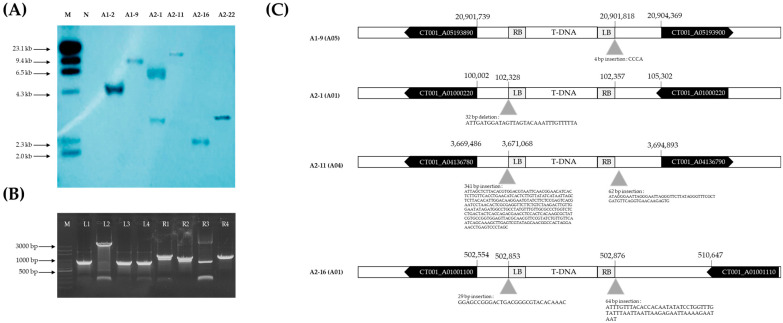
Analysis of T-DNA copy number and insertion position in E_0_ *AGAMOUS-like (AGL)*-edited lines. (**A**) Southern hybridization analysis for identifying the copy number of T-DNA in the E_0_ *AGL*-edited lines’ genome. Briefly, 30 μg of genomic DNA was digested with *Eco*RI, separated, and blotted onto Hybond N^+^ nylon membranes for hybridization with a [^32^P]-labeled probe. The approximate DNA molecular size marker is indicated on the left. M, λ *Hin*dIII molecular ladder; N, negative control; lane, E_0_ *AGL*-edited lines showing late bolting and sequence mutations. (**B**) A modified variable argument thermal asymmetric interlaced PCR (VA-TAIL PCR) analysis to confirm the flanking DNA sequence of T-DNA in the E_0_ *AGL*-edited lines’ genome. Arbitrary degenerate (AD) primers and T-DNA border-specific primers were designed and amplified using 100 ng of genomic DNA as a template. M, 100 bp DNA ladder; lane, E_0_ *AGL*-edited lines. (**C**) Schematic diagram of the T-DNA insertion site in the E_0_ *AGL*-edited lines’ genome. T-DNA was inserted into the intergenic region of the genome of the E_0_ *AGL*-edited lines.

The T-DNA copy number of the E_0_ *AGL*-edited lines was analyzed using Southern hybridization. A 709 bp probe targeting the selection marker, *hpt* gene, was designed and ^32^P-radio-labeled. The control line, ‘CT001,′ was used as a negative control, and the λ *Hin*dIII molecular ladder was loaded for reference. In this study, several signals of 2.0–23.1 kb were observed in the E_0_ *AGL*-edited lines and no signal was observed in the negative control (Figure 6A). This means that the T-DNA was randomly inserted into the genome of the E_0_ *AGL*-edited lines. One copy of T-DNA was inserted into A1-2, A1-9, A2-11, A2-16, and A2-22 line genomes. Three signals were observed in the E_0_ A2-1 line genome. In addition, one copy of T-DNA inserted into the E_0_ *AGL*-edited lines was analyzed for the T-DNA-flanking region using variable argument thermal asymmetric interlaced PCR (VA-TAIL PCR) analysis (Figure 6B). T-DNA was completely inserted into the intergenic region without affecting the expression levels of the other genes (Figure 6C).

### 2.5. Mutagenesis Inheritance Analysis in T-DNA-Free E_1_ AGL-Edited Lines

To select T-DNA-free E_1_ *AGL*-edited lines, PCR analysis was performed using two primer sets targeting partial sequences of hyg^R^ and Cas9hc:NLS:HA (Cas9hc). As a result, T-DNA-free E_1_ *AGL*-edited lines without PCR amplicon were identified. In the A1-2 and A1-9 lines, four and three T-DNA-free E_1_ *AGL*-edited lines were identified, respectively (Figure S1 in Supplementary Materials). Similarly, T-DNA-free E_1_ *AGL24*-edited lines (A2-1-2, A2-1-6, A2-1-10, A2-1-12, A2-11-1, A2-11-6, A2-16-4, A2-16-12, A2-22-1, A2-22-5, A2-22-9, and A2-22-16) without a PCR amplicon were identified in the A2-1, A2-11, A2-16, and A2-22 lines (Figure S2 in Supplementary Materials).

**Figure 7:**
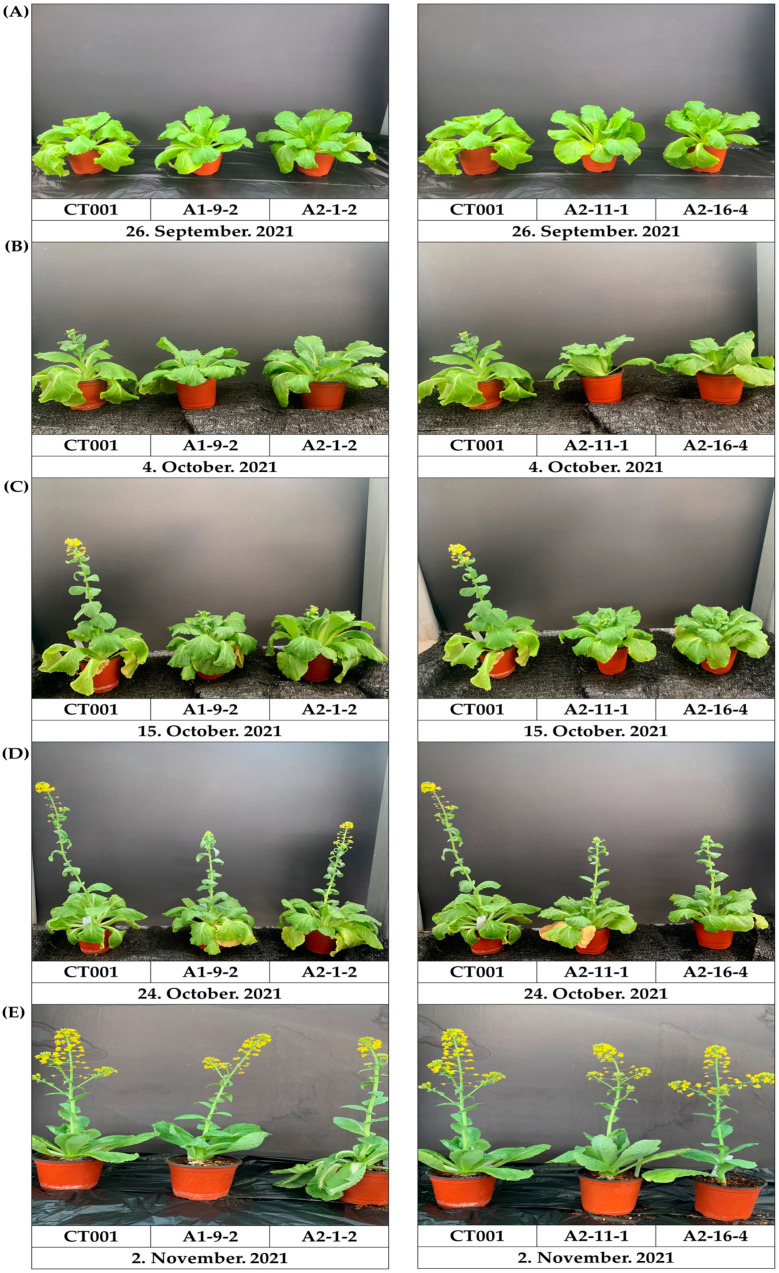
Observation of the bolting and vegetative growth of the inbred line ‘CT001′ and the T-DNA-free E_1_ *AGL*-edited lines. (**A**) Vegetative stage before bolting. (**B**) Beginning stage of bolting. (**C**) Early stage of bolting. (**D**) Middle stage of bolting. (**E**) End stage of bolting. Left, inbred line ‘CT001′; middle and right, T-DNA-free E_1_ *AGAMOUS-like (AGL)*-edited lines.

E_1_ *AGL*-edited lines were grown, and leaves were collected for genomic DNA extraction. PCR and RT-PCR analyses using the same primer sets were performed to confirm that the target sequence mutation remained stable in the T-DNA-free E_1_ *AGL*-edited lines (Figures S1 and S2 in Supplementary Materials). Mutagenesis patterns were compared between E_0_ and E_1_ gene-edited lines. In the E_1_ *AGL*-edited lines, some individual lines maintained the same sequence as the E_0_ *AGL*-edited lines; however, some did not inherit the sequence change and showed different sequence mutations. The E_1_ A2-11-1 line inherited mutation patterns in *CT001_A03122450* and *CT001_A01013460* genes, the but E_1_ A2-11-6 line showed a changed mutation pattern in the *CT001_A01013460* gene (Table 1). Seven nucleotides containing the PAM sequence were deleted, and a single nucleotide was observed for nucleotide transversion. In addition, the *CT001_A03122450* gene in E_1_ A2-16-4 and A2-16-12 lines showed the inheritance of sequence mutations, whereas the *CT001_A01013460* gene showed different mutation patterns. Similarly, the E_1_ A2-1-2 line inherited mutation patterns in the *CT001_A01013460* gene but not in the *CT001_A03122450* gene. After vernalization treatment, the bolting stage was observed in the ‘CT001′ and E_1_ *AGL*-edited lines. On average, it was observed that the bolting and first flower anthesis times of the E_1_ *AGL*-edited lines were 11 days later than those of ‘CT001′ under normal cultivated conditions (Figure 7). As observed in the E_0_ *AGL*-edited lines, the growth process did not show any difference from inbred ‘CT001,′ and it was confirmed that the stem length was restored to a length similar to that of ‘CT001.′ As a result of analyzing the T-DNA-free E_1_ *AGL*-edited lines, the mutagenesis pattern was maintained in some T-DNA-free E_1_ *AGL*-edited lines or a new mutation occurred in some T-DNA-free E_1_ *AGL*-edited lines. However, it was confirmed that the late-bolting trait was inherited in all of the T-DNA-free E_1_ *AGL*-edited lines.

## 3. Discussion

*AGL* genes related to flowering have been studied in various crops to analyze their function. The *AGL17* gene is associated with flower development in the photoperiod pathway. The 35S::AGL17 transgenic *Arabidopsis* plant exhibited an early flowering phenotype, whereas the *agl17* mutant exhibited late flowering. The expression levels of *LEAFY* (*LFY*) and *APETALA1* (*AP1*), which are floral meristem identity genes, decreased in *agl17-1* [27]. *LFY* directly activates *AP1*, which plays dual roles: it specifies the floral meristem, and it acts as a class A gene to determine the identity of perianth organs [28,29]. Activation of the class B gene APETALA3 (AP3), which determines petals and stamens, requires the concerted action of *LFY*, *AP1*, and *UNUSUAL FLORAL ORGANS*, an F-box gene [30,31].

*LFY* also cooperates with a homeobox gene, *WUSCHEL* (*WUS*), to activate the *AG* gene, a type of class C gene that specifies the identity of stamens and carpels [32,33]. *AGL6* is involved in the flowering pathway. The 35S::AGL6 transgenic plants appear to have short vegetative development and early flowering with abnormal phenotype observations in the flower structure [34,35,36]. The *AGL28* gene regulates floral promoters, including *FLOWERING CONTROL LOCUS A* (*FCA*) and *LUMINIDEPENDENS* (*LD*). Overexpression of *AGL28* results in early flowering by upregulation of *FCA* and *LD* [37]. In particular, *AGL24* has been studied in relation to the floral integrator *SUPPRESSOR OF OVEREXPRESSION OF CONSTANS 1* (*SOC1*) gene. The *agl24* mutant showed abnormal phenotypes, such as late flowering and late floral organ development, similar to those of the *SOC1* mutant. *AGL24* expression is correlated with various flowering pathways.

Flowering plants are affected by various flowering pathways, including the circadian rhythm, photoperiod, autonomous, vernalization, gibberellin, flowering time, and meristem identity pathways [38]. The transition from the vegetative to the reproductive stage occurs through the interaction of flowering-related genes. *FLOWERING LOCUS C* (*FLC*), a key regulator gene in flowering, encodes a MADS-box protein that acts as a floral transition repressor, and is downregulated during cold treatment. The *FLC* gene suppresses floral integrator genes, such as *FLOWERING LOCUS T* (*FT*) and *SOC1* [39,40].

This study showed that *AGL19* and *AGL24* directly or indirectly interact with floral integrators, such as *FLC* and *SOC1.* To generate *AGL*-edited Chinese cabbage lines, AGL19_sgRNA and AGL24_sgRNA were designed for specific regions of *AGL* genes and cassettes were ligated with the pHAtC vector. Using conserved sequences, one sgRNA can target and knock out multiple homologous genes. For example, sgRNAs were designed to target the conserved sequences among three gene families (*BnaRGA*, *BnaDA2*, and *BnaFUL*) in *Brassica napus* [41]. *GmPDS*, *GmFAD2*, and *GmALS*, which contain two homologous genes and share 90% homology of the amino acid sequence, were modified using one sgRNA targeting the common sequence of each homologous gene in *Glycine max* [42]. One sgRNA can mutate into three 55 kDa *PP2A B regulatory subunit* (*PR55/B*) homologous genes and generate frameshift-triggered self-compatibility in *Brassica rapa* [43]. These results showed that one conserved sgRNA is sufficient for the development of gene-edited crops. In this study, sgRNAs were designed to target multiple homologous genes by using a conserved sequence (Figure 1). Even with CRISPR/Cas9, non-homologous end joining is sufficient to generate random mutations, including nucleotide insertions, deletions, and transversions. Loss of function in *AGL*-edited lines exhibited a delayed bolting phenotype than did the inbred line ‘CT001′ (Figure 3). The occurrence of an indel mutation in the *AGL19* and *AGL24* genes creates an early stop codon, resulting in the loss of function of the AGL protein. Furthermore, the occurrence of frameshift mutations caused structural transitions and introduced an early stop codon in the AGL19 and AGL24 proteins (Figure 5). The E_0_ *AGL*-edited line with a 1 bp indel mutation and frameshift mutation exhibited late flowering. Similarly, various gene-edited lines have been developed using CRISPR/Cas9. The frameshift in the *VERNALIZATION1* (*VRN1*) gene generates an early stop codon, resulting in the translation of an incomplete protein. Therefore, *BrVRN1* cannot function to suppress *FLC*, and the edited plant shows late flowering [42]. *PR55/B*-edited plants show self-compatibility by producing early stop codons and loss of protein function [43]. These gene-edited plants were advanced to the next generation, and an identical phenotype was observed.

Occasionally, the mutation patterns of target genes in gene-edited plants change during advancement. In this study, the mutation patterns in the *CT001_A01013460* gene of E_1_ A2-11-6, A2-16-4, and A2-16-12 lines were found to be different from those of the E_0_ A2-11 and A2-16 lines. Using the CRISPR/Cas9 system, a 1 bp insertion was observed in the A2-11 line, but a 1 bp transversion and a 7 bp deletion appeared in the A2-11-6 line. In addition, a 6 bp deletion was confirmed in the *CT001_A01013460* gene of the A2-16-4 and A2-16-12 lines. In previous studies, different InDeL mutations of the target region in next generations have been reported for rice [44,45]. The coding sequences of five wheat genes were targeted using the CRISPR/Cas9 system. Among them, only a single 1 bp insertion and deletion, respectively, were detected in the *TaDA2* gene of the T_0_ plant, but more variations were detected in the T_1_ generation [46]. Additional mutations were created for the next generation. Only a 1 bp insertion mutation was observed in the *AOX* gene in T_0_ rice plants, whereas additional mutations were found in the progeny lines [47]. Mutation patterns using CRISPR/Cas9 can be inherited or lost in progeny lines. The occurrence of somatic variations can affect the mutation types. In addition, different plant tissues have been confirmed to have different mutation patterns [48].

Finally, transgene-free plants were secured and useful phenotypes were maintained (Figure 7). As the corresponding T-DNA is removed, analysis of any changes in the surrounding base sequence for the T-DNA-free E_1_ *AGL*-edited lines showing late bolting will be needed. Additionally, it is necessary to identify E_2_ *AGL*-edited lines that exhibit a homozygous mutation pattern via next-generation progression. Developing transgenic plants using traditional techniques for inserting external genes has gotten tangled with the issue surrounding genetically modified (GM) crops, namely the retention of T-DNA in the plant genome to obtain a useful transgenic plant phenotype. However, T-DNA removal is possible through generation advancement in gene-edited plants developed using the CRISPR/Cas9 system. Thus, we believe that the CRISPR/Cas9 system is a useful technique for the development of gene-edited transgene-free plants.

## 4. Materials and Methods

### 4.1. sgRNA Design and Construction of Gene-Editing Vectors

Two *AGL* genes were selected to delay the bolting stage of *Brassica rapa* ssp. *pekinensis*. sgRNA design and vector construction were performed, as described previously [41]. sgRNAs were designed to target the *AGL19* and *AGL24* genes after counting the number of each homologous gene in the ‘CT001′ genome using FSTVAL (http://bioinfo.mju.ac.kr/fstval/; accessed on 4 February 2019). sgRNAs were designed with 23 nucleotides composed of a 20 bp random guide sequence and a 3 bp NGG sequence (N can be A, T, G, or C) using CRISPR direct (http://crispr.dbcls.jp/; accessed on 6 February 2019). To modulate *AGL19* and *AGL24* gene expression, candidate sgRNAs were selected from the exonic region, and a low off-targeting frequency was confirmed based on the ‘CT001′ genome using the BioEdit program (http://en.bio-soft.net/format/BioEdit.html; accessed on 12 February 2019). To compare similar sequences, sgRNA sequences were blasted to the whole genome sequence using the inbred line ‘CT001′ pseudomolecule sequence as a reference. As a result, it was expected that other genes were not targeted by the designed sgRNAs.

To delay the bolting stage, both sgRNAs were designed to target two homologous genes, *AGL19* and *AGL24*. AGL19_sgRNA was designed to target the fourth exon of A03 and the second exon of A08. AGL24_sgRNA was designed to target the first exons of A01 and A03. The constructed gene-editing vectors (A1 and A2) containing the hygromycin phosphotransferase (*hpt*) gene as a selection marker were transferred into *Agrobacterium tumefaciens* LBA4404 using a modified freeze-thaw method [49].

### 4.2. Development of AGL-Edited Chinese Cabbage Lines and Selection Using PCR Analysis

The inbred line ‘CT001’ was used to develop *AGL*-edited Chinese cabbage lines. The seeds of ‘CT001’ were sterilized and sown in MS basal medium in vitro. *AGL*-edited lines were generated using *Agrobacterium*-mediated transformation. The infected explants were transferred to the selection medium with hygromycin every 2 weeks until shoots emerged. After root development, the tentative *AGL*-edited lines were cultivated in a culture room at Kyung Hee University (Yongin, Korea).

The selection of tentative *AGL*-edited lines was performed by confirming T-DNA insertion. The genomic DNA (gDNA) of ‘CT001’ and tentative *AGL*-edited lines was used as a template, and PCR analysis was conducted using the 2× H-Star *Taq* PCR master mix (Biofact, Seoul, Korea). Two primer sets were designed to target the partial sequences of the hyg^R^ and Cas9hc regions (Table S1 in Supplementary Materials). The PCR conditions were as follows: initial denaturation at 95 °C for 15 min; 35 cycles of 95 °C for 30 s, 60 °C for 20 s, and 72 °C for 45 s; and a final extension step at 72 °C for 5 min. PCR amplicons were visualized using electrophoresis on 1% agarose gel.

To confirm the mutations of the *AGL19* and *AGL24* gene sequence, total RNA was extracted from the leaves using the P&C Rapid RNA Prep Kit (Biosolution, Suwon, Korea). The purity and concentration of the extracted RNA were checked using a Nanodrop^®^ ND-1000 spectrophotometer (NanoDrop Technologies, Wilmington, SA, Australia), and cDNA was synthesized using the LyoFACT™ RT Pre-Mix (Biofact, Seoul, Korea). The cDNA synthesis conditions were as follows: reaction at room temperature for 5 min, reverse transcription at 50 °C for 1 h, and RNase inactivation and extension at 95 °C for 10 min. Specific primer sets were designed to target each exonic region of homologous genes (Table S1 in Supplementary Materials). The cDNA of ‘CT001’ and *AGL*-edited lines was used as a template, and RT-PCR analysis was conducted using the 2X H-Star *Taq* PCR master mix (Biofact, Seoul, Korea). RT-PCR conditions were as follows: initial denaturation at 95 °C for 15 min; 35 cycles of 95 °C for 30 s, 58 °C for 20 s, and 72 °C for 20 s; and a final extension step at 72 °C for 5 min. RT-PCR amplicons were eluted using the P&C Multiple Elution Kit (Biosolution, Suwon, Korea) and sequenced using BTSeq (Celemics, Seoul, Korea). The obtained sequence data were aligned and translated into amino acid sequences.

T-DNA-free E_1_ *AGL*-edited lines were selected using PCR analysis with genomic DNA from 3-week-old E_1_ seedlings and T-DNA-specific primers sets. Lines without the PCR amplicon were identified as T-DNA-free E_1_ *AGL*-edited lines.

### 4.3. Bolting Time Record and Stem Length Measurement of Selected AGL-Edited Lines

To examine the bolting time of *AGL*-edited lines, ‘CT001’ and *AGL*-edited lines were randomly placed and artificially vernalized in a cold room for 7 weeks at 4 °C under a 16 h/8 h light/dark photoperiod. After vernalization, the lines were placed in a greenhouse at 23 °C, and then the bolting date and stem length were recorded. The number of days between the emergence and appearance of the first floral axis of each *AGL*-edited line was confirmed. The selected E_0_ *AGL*-edited lines were self-pollinated and advanced to the E_1_ generation.

### 4.4. Identifying the Number of T-DNA Copies and Insertion Site

Southern hybridization was conducted to determine the T-DNA copy number in the genome of E_0_ *AGL*-edited lines. gDNA was cut using *Eco*RI at several recognition sites inside the ‘CT001’ genome [43]. Digested gDNA and a λ *Hin*dIII molecular marker were loaded onto 1% agarose gel, and electrophoresis was conducted at 45 V for 7 h. Denatured DNA was transferred onto a Hybond N^+^ nylon membrane (Amersham Pharmacia, Buckinghamshire, UK). The probe was designed using a 709 bp of the *hpt* gene of the T-DNA, and the eluted PCR amplicon was labeled with ^32^P-dCTP using the BcaBEST labeling kit (TaKaRa, Otsu, Japan). The nylon membrane and labeled probe were hybridized and washed in a shaking incubator at 60 °C. They were then transferred into a cassette for exposure to an X-ray film and visualized.

To confirm the T-DNA insertion site of the E_0_ *AGL*-edited lines, modified variable-argument–thermal asymmetric interlaced PCR (VA-TAIL PCR) was performed [50]. To develop an effective VA-TAIL PCR method, Chinese-cabbage-specific arbitrary degenerate (AD) primers were selected (Table S2 in Supplementary Materials). In addition, four primers were designed using Vector NTI software (Invitrogen, Carlsbad, CA, USA) that could bind to the left and right borders of the pHAtC-AGL vector introduced into the gene-edited line (Table S3 in Supplementary Materials). In addition, to increase the specificity of the reaction, 5–6 bp of the 3′ end of each primer were overlapped to match the 5′ end of the primer in the next step. The total reaction volume was 30 μL and contained 100 ng of gDNA, AD primers, border-specific primers, and dH_2_O. VA-TAIL PCR analysis was performed using the 2X *Taq* PCR Master Mix (Biofact, Seoul, Korea) with a thermocycler (Applied Biosystems, Carlsbad, CA, USA) programmed as shown in Table 2. Specific PCR amplicons were eluted and sequenced using Celemics (Seoul, Korea). Finally, the flanking DNA sequence (FDS) was analyzed to confirm its homology with the T-DNA sequence. The chromosome (numbered A01–A10) bearing the T-DNA insert was identified, and the positions of insertion (intergenic, exon, intron, 5′ upstream-1000, and 3′ downstream-300) of the T-DNA in the respective chromosomes were analyzed.

## 5. Conclusions

This is the first study to explore the development of *AGL*-edited Chinese cabbage lines with a late-bolting phenotype using the CRISPR/Cas9 system and targeting the *AGL19* and *AGL24* genes. To target specific regions, sgRNA sequences were designed with a low off-targeting probability, and gene-editing vectors were constructed. Late-bolting *AGL*-edited lines were developed, and target sequence mutations were analyzed. Finally, elite lines with one copy of T-DNA inserted into the intergenic region were obtained. Therefore, it was possible to develop late-bolting Chinese cabbage lines by targeting the *AGL* genes using the CRISPR/Cas9 system. The results of this study are expected to be used as basic data for developing crops with useful traits for responding to climatic changes using the CRISPR/Cas9 system.

## Figures and Tables

**Table 1:** Mutagenesis patterns in T-DNA-free E_1_ *AGL*-edited lines.

E_0_ Gene-Edited Line	E_1_ Gene-Edited Line	Chr. ^z^	Target Gene	Mutation Type ^y^
A1-2	A1-2-5	A03	*CT001_A03121400*	–
		A08	*CT001_A08282630*	–
A1-9	A1-9-2	A03	*CT001_A03121400*	1 bp In (T)
		A08	*CT001_A08282630*	–
A2-1	A2-1-2	A03	*CT001_A03122450*	60 bp Del
		A01	*CT001_A01013460*	1 bp Del (T)
A2-11	A2-11-1	A03	*CT001_A03122450*	1 bp In (A)
		A01	*CT001_A01013460*	1 bp In (C)
	A2-11-6	A03	*CT001_A03122450*	1 bp In (A)
		A01	*CT001_A01013460*	1 bp Tv, 7 bp Del
A2-16	A2-16-4	A03	*CT001_A03122450*	1 bp In (C)
		A01	*CT001_A01013460*	6 bp Del
	A2-16-12	A03	*CT001_A03122450*	1 bp In (C)
		A01	*CT001_A01013460*	6 bp Del
A2-22	A2-22-5	A03	*CT001_A03122450*	–
		A01	*CT001_A01013460*	120 bp Del

^z^ Chromosome numbers. ^y^ Tv, transversion; Del, deletion; In, insertion.

**Table 2:** Modified variable-argument–thermal asymmetric interlaced polymerase chain reaction (VA-TAIL PCR) conditions used in this study.

Reaction	Number of Cycles	Thermal Settings
Primary (AD-LSP1/RSP1) ^z^	1	95 °C 2 min
	5	94 °C 30 s, 72 °C 4 min
	2	94 °C 30 s, 25 °C ramping to 72 °C in 4 min
	15	94 °C 30 s, 72 °C 4 min 94 °C 20 s, 72 °C 4 min 94 °C 30 s, 44 °C 1 min, 72 °C 2 min 30 s
	1	72 °C 5 min, 16 °C hold
Secondary (AD-LSP2/RSP2)	1	95 °C 2 min
	5	94 °C 20 s, 72 °C 4 min
	15	94 °C 30 s, 72 °C 4 min 94 °C 20 s, 72 °C 4 min 94 °C 30 s, 44 °C 1 min, 72 °C 2 min 30 s
	5	94 °C 20 s, 44 °C 1 min, 72 °C 2 min 30 s
	1	72 °C 5 min, 16 °C hold

^z^ AD, arbitrary degenerate primers; LSP, left-border specific primer; RSP, tight-border specific primer.

## Data Availability

Not applicable.

## Supplementary Materials

The following are available online at https://www.mdpi.com/article/10.3390/ijms232315009/s1.
