# Supplementary material for: CRISPR/Cas9-Mediated Editing of AGAMOUS-like Genes Results in a Late-Bolting Phenotype in Chinese Cabbage (Brassica rapa ssp. pekinensis)
Source: Int J Mol Sci. 2022 Nov 30;23(23):15009. doi: 10.3390/ijms232315009 (PMC9735848; doi:10.3390/ijms232315009)
Supplement: Supplementary file 1 [file ijms-23-15009-s001.zip › ijms-2048176-supplementary.pdf]

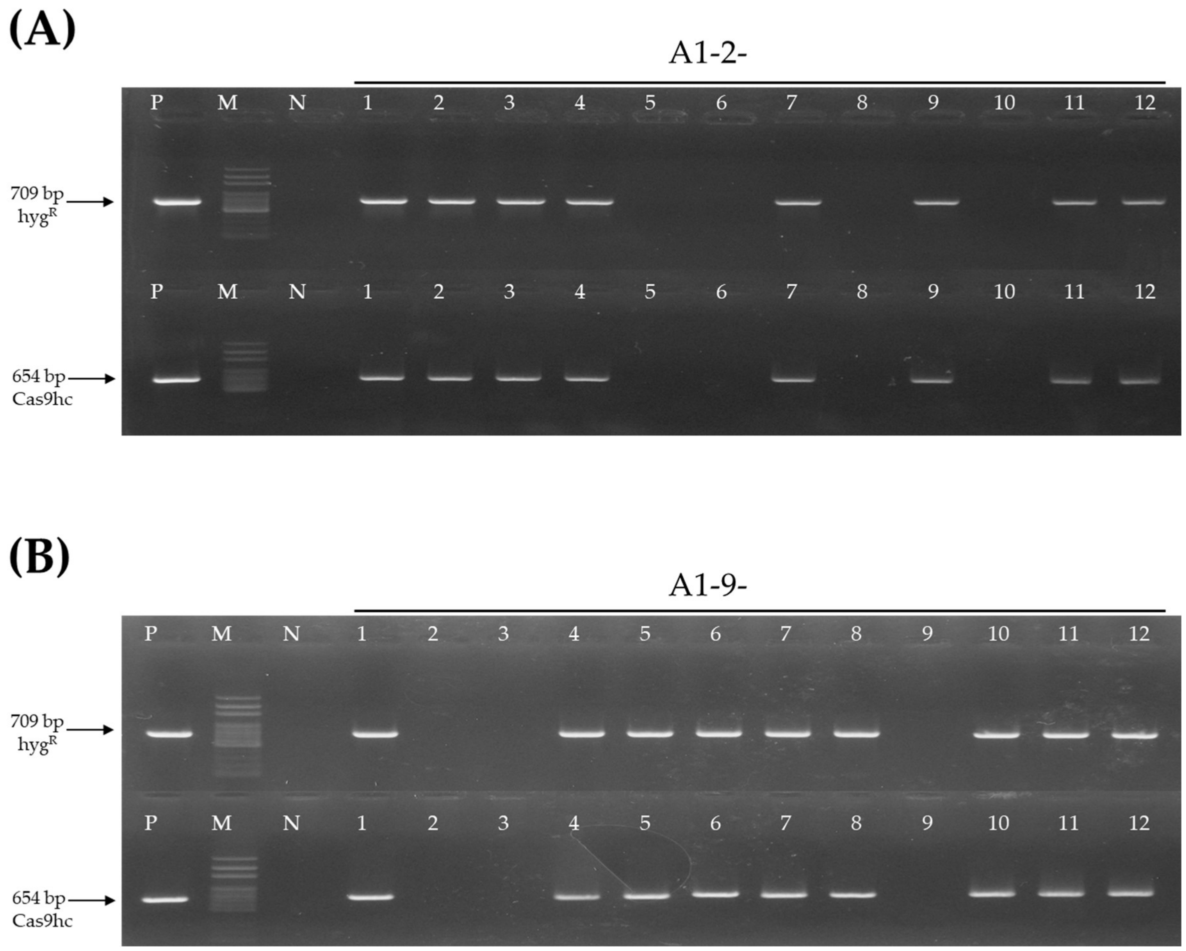

Figure S1. Selection of E_1 *AGL19*-edited lines using polymerase chain reaction (PCR) analysis. (**A**) PCR analysis with hyg^R and Cas9hc primer sets of E_1 A1-2-edited lines. (**B**) PCR analysis with hyg^R and Cas9hc primer sets of E_1 A1-9-edited lines. The 709 bp and 654 bp expected PCR products are indicated with an arrow, respectively. P, positive control; M, 100 bp DNA ladder; N, negative control; Numbering lane, gene-edited lines.

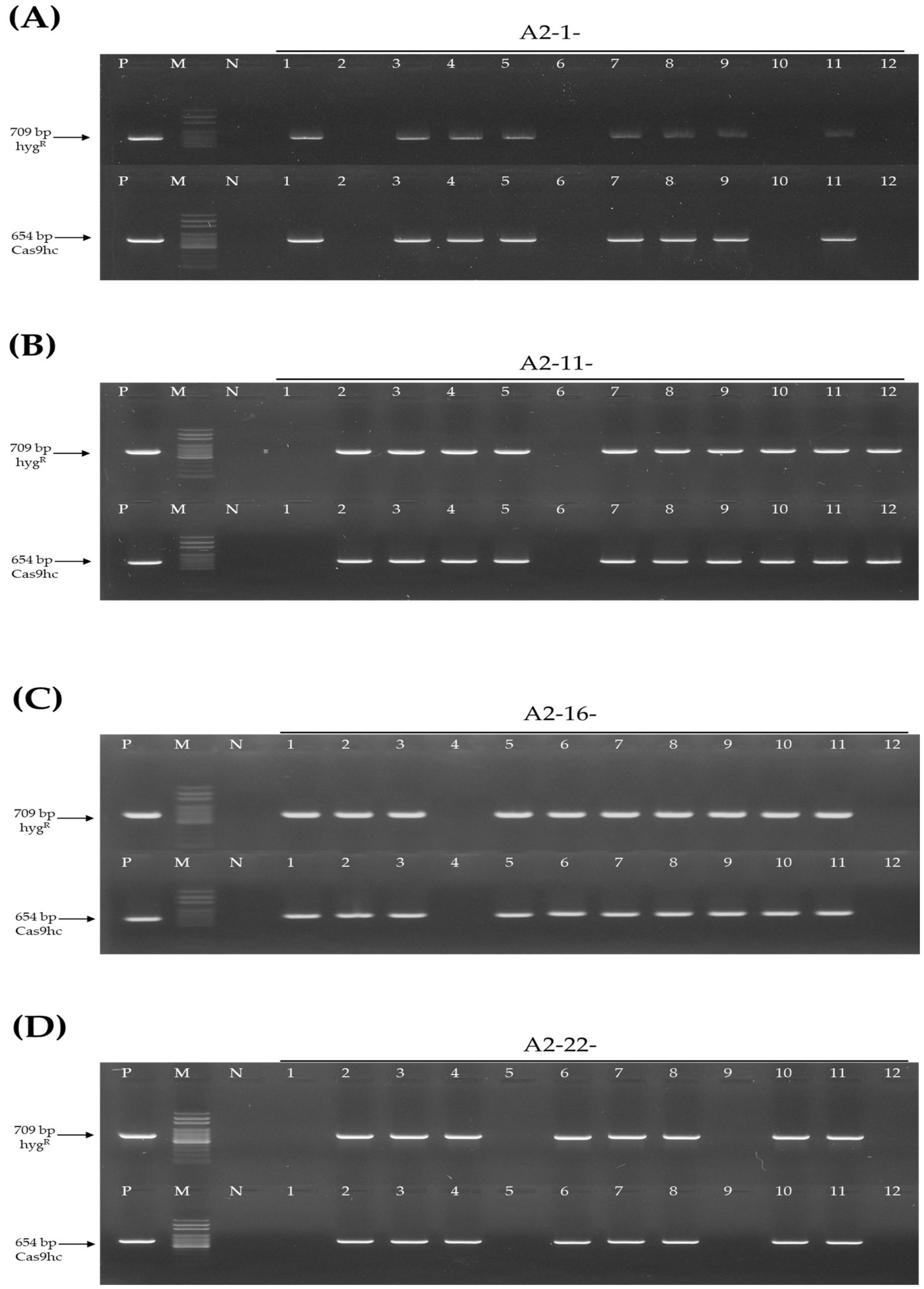

Figure S2. Selection of E_1 *AGL24*-edited lines using polymerase chain reaction (PCR) analysis. (**A**) PCR analysis with hyg^R and Cas9hc primer sets of E_1 A2-1-edited lines. (**B**) PCR analysis with hyg^R and Cas9hc primer sets of E_1 A2-11-edited lines. (**C**) PCR analysis with hyg^R and Cas9hc primer sets of E_1 A2-16-edited lines. (**D**) PCR analysis with hyg^R and Cas9hc primer sets of E_1 A2-22-edited lines. The 709 bp and 654 bp expected PCR products are indicated with an arrow, respectively. P, positive control; M, 100 bp DNA ladder; N, negative control; Numbering lane, gene-edited lines.

Table S1. List of primer sets for polymerase chain reaction (PCR) analysis.

Name	Primer	Sequence (5'→3')	Expected product size (bp)
**Hyg^R**	F^z	CGT CTG CTG CTC CAT ACA AG	709
	R	TGT CGA GAA GTT TCT GAT CGA	
**Cas9hc**	F	CCG CCA GGA GGA CTT CTA CC	654
	R	ATG TTC TCG GGC TTG TGG CG	
**CT001_A03121400**	F	GGA GTT TCT TGG ATT GTC TTG G	297
	R	GTT GTC AAA ATC TCA GGA GAG G	
**CT001_A08282630**	F	GAA GAA ATT GAT AAG CTG AAG	476
	R	TTT CTT GGA TTG TCT TGG CT	
**CT001_A03122450**	F	AAA CCA CAC ATG CAA AGT CG	1381
	R	GCT GAT GAA CTT TCG GTT CT	
**CT001_A01013460**	F	ATG GCG AGA GAG AAG ATA AG	318
	R	CTC CGA GCC CAA GAA TAA ATT	

^{z}: F, forward primer; R, reverse primer.

Table S2. List of degenerate primers for variable argument-thermal asymmetric interlaced polymerase chain reaction (VA-TAIL PCR) analysis.

Target domain	Name	Sequence (5'→3')	Variable argument[z]
**Zinc finger protein LSD1**	**BrAD1**[y]	GAM RTG NCT VAM WTT G[x]	192
	**BrAD2**	DTA ASA TGN HNT TGC T	288

[z] : The number of primer combinations of AD primers.

[y] : BrAD primers are degenerate primers.

[x] : Mixed bases. M = A/C; R = A/G; V : A/C/G; W : A/T; D = A/G/T; S : G/C; H = A/C/T; N = any base.

Table S3. List of nested long T-DNA-specific primers for variable argument-thermal asymmetric interlaced polymerase chain reaction (VA-TAIL PCR) analysis.

Primer	Sequence (5'→3')	GC%	TM(℃)
LSP1	ATA GTG GAA ACC GAC GCC CCA GCA[z]	58.3	66.6
LSP2	CCA GCA CTC GTC CGA GGG CAA AG	65.2	66.8
RSP1	AAA GTA TAC CCC TAC GAC GTG CCC G	56.0	63.8
RSP2	GCC CGA CTA CGC CTA ACA CCC AG	65.2	66.2

[z] : Underlined nucleotide sequences indicate the overlapping sequence.
